# ADNP: in search for molecular mechanisms and innovative therapeutic strategies for frontotemporal degeneration

**DOI:** 10.3389/fnagi.2015.00205

**Published:** 2015-10-29

**Authors:** Illana Gozes, Yanina Ivashko-Pachima

**Affiliations:** Elton Laboratory for Molecular Neuroendocrinology, Department of Human Molecular Genetics and Biochemistry, Sackler School of Medicine, Sagol School of Neuroscience & Adams Super Center for Brain Studies, Tel Aviv UniversityTel Aviv, Israel

**Keywords:** acitivity-dependent neuroprotective protein (ADNP), NAP (davunetide), microtubules, Alzheimer's disease, neuroprotection

## Abstract

Activity-dependent neuroprotective protein (ADNP) is deregulated in Alzheimer's disease (AD) and in schizophrenia and mutated in autism. In mice, ADNP is essential for brain formation and ADNP haploinsufficiency is associated with cognitive and social deficits and tauopathy. Tauopathy, a major pathology in AD, is also found in ~45% of frontotemporal dementias (FTDs). Tau transcript, a product of a single gene, undergoes alternative splicing. Tau splicing seems to be altered in FTD brain. In transgenic mice overexpressing a mutated tau in the cerebral cortex, significant increases in ADNP transcript expression were observed in the cerebral cortex of young transgenic mice (~disease onset) and a marked decrease with aging as compared to control littermates. ADNP is a member of the SWItch/Sucrose NonFermentable (SWI/SNF) chromatin remodeling complex also associated with alternative splicing, including tau transcript splicing. Further cellular interactions of ADNP include association with microtubules, with tau being a microtubule—associated protein. NAP (davundetide), a novel drug candidate derived from ADNP, increases ADNP-microtubule association and protects against tauopathy and cognitive deficiencies in mice. Although, NAP did not provide protection in progressive supranuclear palsy (PSP), a pure tauopathy, it increased cognitive scores in amnestic mild cognitively impaired patients and protected functional activity in schizophrenia patients. This mini-review focuses on ADNP in the context of FTD and tau/microtubules and proposes NAP as a novel drug target for future clinical evaluations.

## Activity-dependent neuroprotective protein (ADNP) deregulation is linked to brain diseases

Activity-dependent neuroprotective protein (ADNP) that has been discovered in our laboratory (Bassan et al., 1999; Zamostiano et al., 2001) has been associated with various diseases such as Alzheimer's disease (AD)—with ADNP protein levels suggested to be decreased in patient serum samples compared to controls (Yang et al., 2012), schizophrenia—with ADNP mRNA levels increased in lymphocytes of patients compared to controls (Merenlender-Wagner et al., 2015), multiple sclerosis—with ADNP mRNA levels decreased in nucleated blood cells of patients (Braitch et al., 2009), mutated in autism and associated with cognitive deficits (O'Roak et al., 2012a,b; Helsmoortel et al., 2014; Gozes et al., 2015a), and mutated in cancer (Zamostiano et al., 2001). Additional studies identified ADNP single nucleotide polymorphism in the second intron, upstream the protein coding region, as a risk factor to prostate cancer (Al Olama et al., 2014). Our recent bioinformatics showed that ADNP is unique to chordata, a gene shaping the brain of higher organisms (Gozes et al., 2015b). In mice, ADNP is essential for brain formation (Pinhasov et al., 2003; Mandel et al., 2007) and ADNP haploinsufficiency is associated with cognitive and social deficits and tauopathy (Vulih-Shultzman et al., 2007). Here we posit an association of ADNP with frontotemportal degeneration/dementia (FTD).

## Pathophysiology of FTD

FTD is a spectrum of neurodegenerative diseases with pathological involvement of the frontal and temporal lobes, which refers to a variety of clinical manifestations of frontotemporal lobar degeneration (FTLD). FTD, the most common subtype of FTLD, is also divided into two main syndromes (Hopkins and Chan, 2015): a behavioral variant (bvFTD), characterized by changes in social and personal conduct with loss of volition and abstract thought, as well as decreased speech output; and primary progressive aphasia (PPA), characterized by a speech and language impairment resulting in mutism and an inability to communicate. PPA is further divided into two syndromes, the agrammatic variant and semantic dementia (Benussi et al., 2015). In certain forms of FTD, parkinsonian symptoms and amyotrophic lateral sclerosis (ALS)-like motor abnormalities may be present. In addition to FTLD subtypes, there are different clinical syndromes [corticobasal degeneration (CBD), progressive supranuclear palsy (PSP), and motor neuron diseases] with overlapping clinical/pathological features, leading to confusion in the terminology and to difficulties in diagnosis (Mendez et al., 2007; Rascovsky et al., 2011). Another classification of FTLD subtypes uses neuropathological findings. Mackenzie et al. (2009, 2010), classified two main neuropathological subtypes of FTLD: FTLD with tau-positive inclusions (FTLD-tau), caused by tau mutations or tau deregulation (Gozes, 2010), and FLTD with ubiquitinated inclusions (FTLDU), caused by mutations/deregulation in the TARDBP, GRN, VCP, and CHMP2B genes. A recent review extends these definitions and describes, in depth, the network of RNA and protein interactions in FTD (Fontana et al., 2015), encompassing C9ORF72, a gene mutated in FTD and ALS (DeJesus-Hernandez et al., 2011; Renton et al., 2011) and associated with RNA splicing factors (Cooper-Knock et al., 2015), which in turn may regulate tau. Thus, potential links between C9ORF72 to tau deserve future studies. Interestingly, recent findings identified C9ORF72 repeat expansions in 4 of 334 subjects [1.2% (or 1.8% of 217 tested families)]. All the tested subjects had behavioral phenotypes and also harbored well-known pathogenic mutations in either progranulin (GRN: p.C466LfsX46, p.R493X, p.C31LfsX35) or tau (MAPT: p.P301L), contributing to the pleiotropy of the disease (van Blitterswijk et al., 2013).

## ADNP expression is correlated with the microtubule associated protein tau that is in turn linked to FTD

The neurodegeneration underlying FTD results from cortical and subcortical neuronal loss in the frontal and temporal lobes, whereas tau-positive inclusions may be found in ~45% of FTLDs (Boxer et al., 2013). Tau transcript, a product of a single gene, undergoes alternative splicing. Tau splicing seems to be altered in FTD brain. Alternative splicing around exon 10 of the tau transcript yields tau protein variants including tau protein containing either 3 or 4 microtubule binding repeat domains (tau 3R or 4R), associated with dynamic and stable microtubules, respectively (Goedert et al., 1988, 1989a,b; Goedert and Jakes, 1990). The healthy human brain exhibits a 1/1 ratio of tau 3R/4R and deviation from this ratio are pathological features of FTD taupathies (Goedert et al., 1988, 1989a,b; Hutton et al., 1998; Spillantini et al., 1998; Goedert and Spillantini, 2011).

In FTD mouse model overexpressing a mutated tau (4R) in the cerebral cortex [rTg(tauP301L)4510], significant transient increase in ADNP transcript expression (~three-fold) was observed in young transgenic mice at the disease onset compared to control littermates. This effect has not been seen in the cerebellum that is lacking an expression of a mutated tau. The increase in ADNP paralleled an increase in tau 3R and blocking the mutated tau 4R transgene expression resulted in normalization of ADNP and tau 3R mRNA levels. Furthermore, an aging-augmented decrease in ADNP expression was observed in the in the cerebral cortex of the mutated mice, compared to control littermates (Gozes et al., 2014a; Schirer et al., 2014). These findings suggest an ADNP/tau/FTD interaction.

Mechanistically, we have shown that ADNP is a member of the SWItch/Sucrose NonFermentable (SWI/SNF) chromatin remodeling complex (Mandel and Gozes, 2007). As such, ADNP binds to heterochromatin protein 1 (Mandel et al., 2007; Mosch et al., 2011). Brahma (Brm), a component of the SWI/SNF complex regulating alternative splicing, showed a similar developmental expression pattern to ADNP in the rTg4510 mice. Immunoprecipitations further suggested a Brm-ADNP interaction. Further protein-protein association was found between ADNP and the polypyrimidine tract-binding protein (PTB)-associated splicing factor (PSF), with PSF being a direct regulator of tau transcript splicing (Schirer et al., 2014). These ADNP protein-protein interactions implicate a potential influence of ADNP expression on tau mRNA splicing. Notably, PSF interacts with peroxisome proliferator-activated receptor gamma (PPARc), a nuclear receptor that plays an essential role in cell proliferation, apoptosis, and inflammation (Esteves et al., 2014), serving as a therapeutic target in AD (Roses et al., 2013). PSF regulation and the regulation of tau splice variant expression have both been associated with learning and memory (Antunes-Martins et al., 2007). Furthermore, PSF (also known as SFPQ, splicing factor, proline, and glutamine-rich) is depleted from the nucleus and accumulates in the cytoplasm in AD and in FTLD, in brain areas affected by tau pathology. This cellular localization is mediated by tau protein over-expression (Ke et al., 2012). In this respect, ADNP haploinsufficiency was associated with cognitive and social deficits and aging-associated tau hyperphosphorylation, neurofibrillary tangle-like accumulation, and neurodegeneration, in mice (Vulih-Shultzman et al., 2007).

## ADNP is linked to microtubules and autophagy

Further cellular interactions of ADNP include association with microtubules (Furman et al., 2004; Oz et al., 2014). Specifically, we showed interaction of ADNP with microtubule end binding proteins (EBs) (Oz et al., 2014). Recent findings identified tau as a regulator of the localization and function of EB1 and EB3 in developing neuronal cells (Sayas et al., 2015). Tau is associated with microtubule dynamics and axonal transport, while EB1 is important for axons (Alves-Silva et al., 2012) and EB3 for dendritic spines (Jaworski et al., 2009) and both EB1 and EB3 are important for ADNP-associated neuronal survival (Oz et al., 2014). In this respect, small hairpin RNA ADNP downregulation (~80% robust reduction) paralleled a significant reduction (~50%) in neurite numbers in neuronal-like teratocarcinoma P19 cells (as measured by microtubule associated protein 2 immunoreactivity) (Mandel et al., 2008).

We also could show a direct association of ADNP with microtubule associated protein 1, light chain 3 (in short, LC3), a major component of the autophagosome and a key regulator of autophagy (Merenlender-Wagner et al., 2015). In this respect, autophagy was shown to be reduced in cases of tauopathy (Schaeffer and Goedert, 2012) and microtubule integrity is tightly associated with functional autophagy (Esteves et al., 2014).

Microtubules are also associated with the cellular protein translation process (Ben-Ze'ev et al., 1979), and in this respect, we showed direct ADNP interaction with the eukaryotic translation initiation factor 4E (eIF4E). As this factor is a regulator of autistic phenotype (Gkogkas et al., 2013; Malishkevich et al., 2015), association between ADNP and eIF4E may be underlying some of the behavioral phenotypes observed in FTD.

## Zinc accumulation is associated with tau pathology

There is current evidence for a relative increase in intracellular zinc in vulnerable regions of the AD brain (Frederickson et al., 2005a; Berti et al., 2015). Zinc is involved in signal transmission/transduction across synapses and therefore modulates synaptic transmission and plasticity (Frederickson et al., 2005b). Besides its physiological functions, zinc dyshomeostasis can contribute to neuronal and astrocytic cell death (Koh et al., 1996; Bossy-Wetzel et al., 2004). Phosphorylation of tau regulates its binding to microtubules and is also associated with tau aggregation in disease. Aberrant phosphorylation is a key feature of tau isolated from the brains of individuals with AD and many other diseases exhibiting tau pathology (Lee et al., 2001). One likely tau kinase is glycogen synthase kinase 3β (GSK3β) (Medina and Avila, 2014). It has been shown that abnormal high concentration (up to 250 μM) of zinc induces GSK-3β activation and tau release from microtubules (Boom et al., 2009). In terms of FTD, for example, a truncating copper/zinc superoxide dismutase (SOD1) mutation, p.Gly141X, is associated with clinical and pathologic heterogeneity, including FTLD (Nakamura et al., 2015).

Taking into account that increased cellular levels of zink can induce release of tau from microtubules and thereby impair microtubule stability, ADNP replacement therapy that we propose here may be of benefit to prevent microtubule disruption and pathological aggregation of tau (Divinski et al., 2004, 2006; Oz et al., 2012, 2014). This statement leads us to clinical relevance of ADNP and the active ADNP fragment peptide, NAP (NAPVSIPQ, davuentide) (Bassan et al., 1999).

## Clinical prospects focusing on the ADNP-derived drug candidate NAP in the context of other FTD drug developments

To date, there is no disease-modifying treatment for FTD, affecting the underlying disease process. As for symptomatic treatments, FTD patients exhibit serotonin (Franceschi et al., 2005) and dopamine (Rinne et al., 2002) deficits, therefore the main pharmacological treatments used for FTD are based on the neurotransmitter replacement and on medications used to improve the behavioral symptoms (Litvan, 2001).

There are several preclinical studies in search for potential drug candidates for combating FTD. As mentioned above, the repeat expansion in C9ORF72 causes FTD and ALS (c9FTD/ALS). RNA of the expanded repeat [r(GGGGCC)exp] forms nuclear foci or undergoes repeat-associated non- ATG (RAN) translation, producing “c9RAN proteins” (Ash et al., 2013; Mori et al., 2013; Zu et al., 2013). Bioactive small molecules targeting r(GGGGCC)exp were designed and found to significantly inhibit RAN translation and foci formation in cultured cells expressing r(GGGGCC)66 and neurons transdifferentiated from fibroblasts of repeat expansion carriers, presenting a possible–targeted c9FTD/ALS therapeutic (Su et al., 2014).

Searching clinicaltrials.gov and touching on a few selected examples for clinical intervention, reveals that oxytocin affecting emotion is being tested (ClinicalTrials.gov Identifier: NCT01937013). Studies evaluating FRM-0334, a small molecule inhibiting histodeacetylase (HDAC), (NCT02149160) are being carried out for FTD patients with granulin gene mutations. In PSP patients, young plasma transfusion is being tested (NCT02460731). Similarly, recombinant humanized anti-tau antibody, C2N-8E12 (ABBV-8E12), is tested (NCT02494024). Salsalate, a medication that belongs to the salicylate and non-steroidal anti-inflammatory drug (NSAID) classes, is being evaluated as potential drug target as well (NCT02422485). Finally, TPI 287, an abeo-taxane —a synthetic derivative of the taxane diterpenoid drugs used in cancer therapy (which shows blood brain barrier permeability) is being tested for microtubule stabilization (NCT02133846). Together, these potential therapeutics target behavior as well as tau pathology/microtubule stability and inflammation, with potential of symptomatic/disease modifying effects. It is early to estimate potential success of any of the above listed drug candidates, but the broad range of the different approaches should pave the path to future therapeutic interventions. Given the complexity of FTD, it is possible that combination therapies will be required and given the progressive degenerative nature of the disease (s), early detection and early intervention is required.

Here, we focus on ADNP and its potential involvement in FTD. The drug candidate NAP (Bassan et al., 1999) contains the EB1, EB3, ADNP interaction site (SIP), and increases ADNP-microtubule association, i.e., EB3-ADNP association (Oz et al., 2014). NAP enhances dendritic spine formation in an EB3-depedent manner and the NAP protection against zinc intoxication requires EB1 and EB3 (Oz et al., 2014). We have further shown that NAP enhances ADNP-LC3 interaction (with LC3 being a part of the microtubule associated protein 1, and a key component of the autophagy process; Merenlender-Wagner et al., 2015). This finding is complemented by the observations that NAP protects microtubule integrity in parallel with protection of autophagy (Esteves et al., 2014).

**Figure 1:**
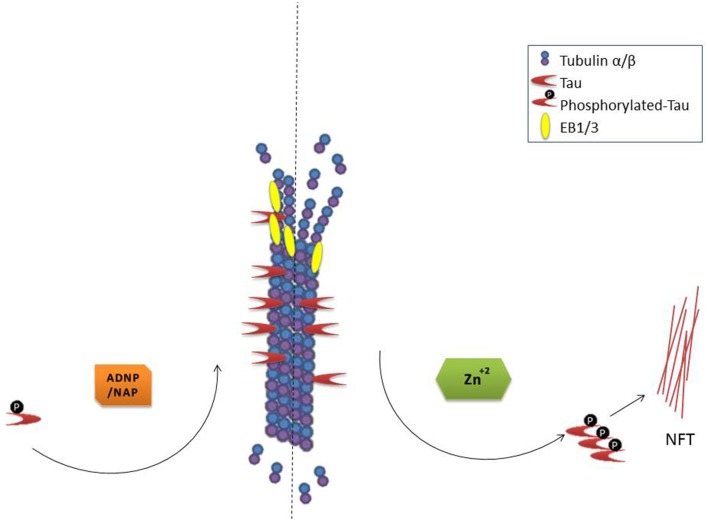
**NAP/ADNP protection of microtubules results in protection against tau pathology (NFT, neurofibrillary tangles)**. Please refer to the text for in depth description.

In cell cultures, NAP protects neurons and astrocytes against ADNP deficiency (Pascual and Guerri, 2007; Vulih-Shultzman et al., 2007), decreased autophagy (Esteves et al., 2014) microtubule disruption, mitochondrial impairment, and apoptosis [e.g., in the presence of excess zinc (Divinski et al., 2004, 2006) or other toxicities (Zemlyak et al., 2009a,b; Idan-Feldman et al., 2012; Esteves et al., 2014)]. Mechanistically, NAP protects against tauopathy *in vitro* (Gozes and Divinski, 2004; Shiryaev et al., 2011; Idan-Feldman et al., 2012) and *in vivo* (Matsuoka et al., 2007, 2008; Vulih-Shultzman et al., 2007; Shiryaev et al., 2009; Jouroukhin et al., 2013; Magen et al., 2014), in part, by enlisting tau back to the microtubules (Shiryaev et al., 2011; Sudo and Baas, 2011; Oz et al., 2012; Quraishe et al., 2013) increasing microtubule dynamics (Oz et al., 2012) and fortifying axonal transport (Jouroukhin et al., 2013; Quraishe et al., 2013). A current working hypothesis for the mechanism by which NAP provides protection is illustrated in Figure 1.

In mice, NAP protects against tauopathy and cognitive deficiencies in a model of ADNP haploinsufficiency (Vulih-Shultzman et al., 2007) and provides protection in transgenic models of FTD (Shiryaev et al., 2009) and AD (Matsuoka et al., 2007, 2008). However, NAP may also be explored further for pathological conditions other than FTD or AD. As NAP provides protection in the SOD1-G93A transgenic mouse model of ALS (Jouroukhin et al., 2013), this therapeutic approach may be considered in the context of ALS. Furthermore, NAP provides protection in the microtubule associated protein 6 (Map6) deficient mouse model of schizophrenia, protecting autophagy and cognitive functions (Merenlender-Wagner et al., 2014).

We have also derived NAP analogs, which provided cognitive as well as protection against tauopathy in mouse models, e.g., NATLSIHQ (NAT; Gozes et al., 2014a), NAP alpha-aminoisobutyric acid (IsoNAP; Gozes et al., 2014b), and all D-amino acids analog, SALLRSIPA (D-SAL; Shiryaev et al., 2011). SAL/D-SAL may fortify NAP protection (Brenneman et al., 2004) and this is a subject of future investigation.

In men, NAP (davunetide) has a safe clinical profile in relatively large cohorts, altogether exposed to >700 subjects. While, NAP (davunetide) did not provide protection in PSP (Boxer et al., 2014), it increased cognitive scores in amnestic mild cognitively impaired patients (Gozes et al., 2009; Morimoto et al., 2013) and protected functional activity in schizophrenia patients (Javitt et al., 2012; Jarskog et al., 2013). We posit that early intervention with NAP will lead to clinical efficacy.

In conclusion, NAP presents an advantage in being a simple small molecule (a short 8 amino acid peptide, derived from a natural essential protein, ADNP) with brain bioavailability through a non-invasive route. However, in this respect, it is similar to oxytocin. Differing from oxytocin, NAP (davunetide) presents a different, unique mechanism of action (interaction with microtubule end binding proteins and increasing ADNP-associated neuroprotection). Thus, the NAP protection of the microtubule structure relies on the fortification of an endogenous process, unlike taxanes or tau immunotherapy. Together these finding envision NAP as an innovative therapeutic approach in FTD.

### Conflict of interest statement

NAP (davunetide) and pipeline products are under term sheet agreement for further clinical development (IG).
